# Structural elucidation of a compound extracted from *Streptomyces Sp.*NLKB45

**DOI:** 10.6026/97320630019820

**Published:** 2023-07-31

**Authors:** B Sudha Kalyani, B Amrutharaj, PS Krishna, K Sreenivasulu

**Affiliations:** IKP-Lifescience Incubator, Hyderabad, Telangana, India; Department of Pharmaceutical Analysis, NIPER, Punjab, India; Lavin Laboratories, Hyderabad, Telangana, India; Department of Biotechnology, KLEF University, Guntur District, A.P., India

**Keywords:** Streptomyces, HPLC, Mass, NMR, structure elucidation

## Abstract

A *Streptomyces Sp.* NLKB45 was isolated from the Nellore coastal region. After checking antimicrobial and antifungal activity the strain was subjected to fermentation in starch casein broth medium. HPLC was used to purify the culture filtrate after it had been extracted with ethyl acetate. The extract showed antibacterial and anticancer properties. The extract was processed and the compound isolated was further analyzed using FTIR, mass spectroscopic method and one- and two-dimensional NMR techniques. Based on the data gathered the compound was identified as 7-((5-methylpyridin-2-ylamino) (phenyl) methyl) quinolin-8-ol with a weight-average molecular weight of about 341 Da.

## Background:

Actinomycetes particularly *Streptomyces Sp*ecies are valuable prokaryotes both economically and biotechnologically. They are responsible for the production of about half of the discovered bioactive secondary metabolites [1], notably antibiotics [1-2], antitumor agents [3], immunosuppressive agents [4] and enzymes [5]. We have reported previously the isolation, optimization of medium conditions for antimicrobial compound production and anticancer studies of indigenous marine actinomycetes strain called NLKB 45 which is showing close relation to *Streptomyces Sp* strain of Accession no (MG241290.1) [6-7]. Therefore, it is of interest to describe the structural elucidation of compound extracted from NLKB45 using NMR studies as described elsewhere [8-9].

## Methodology:

## Instrumentation:

The HPLC system (Agilent 1260 infinity series, Germany) consisted of a G1311C quaternary pump, a G1329B auto sampler, a G1316A column compartment, and a G136D DAD detector operated and data processed with CDS EZ Chrom (OpenLab) software version A.04.05. Separation was achieved under gradient elution on a spherical silica-based Angilent Zobax SBC18 column, 100x9.4 mm, particle size 5.0 mm from USA with a flow rate of 1.0 mL/min at 254 nm and the column temperature was 25°C. The injection volume was 10 µL. The 1D/2D NMR experiments of unknown compound were conducted on a Bruker 400 MHz NMR instrument operated and data processed with Topspin version 3.5 software and Mass spectrometry experiments were performed on a Waters Acquity UPLC-MS with TQD detector (Model G120PD356A), Quaternary solvent manager operated and data processed with Mass Lynx software V 4.1. Shimadzu IR spectrophotometer model IR Prestige-21 operated and data processed with IR Solutions software. Sonicator (Bandellin sonorex), pH meter (Eutech instruments - Cyberscan), Centrifuge Sorvall Lynx4000, N Biotek NB1060 Autoclave, Aditya Scientific Rotary evaporator were used.

## Isolation of marine Actinomycetes:

Mangrove soil samples were taken at the Krishnapatnam port in the Andhra Pradesh region of India's Nellore district. By using the Cross streak technique, the isolated marine actinomycetes were examined for their antogonestic activity towards the bacterial pathogen. The strain that demonstrated the best inhibition against the chosen pathogens was subjected for molecular identification using 16 s rRNA sequencing. A 250 mL capacity Erlenmeyer flask was used for the fermentation process, containing 100 mL of Starch Casein Broth medium to extract crude metabolite by ethyl acetate [6].

## Purification studies of extracted compound:

Sephadex G25 resin was uniformly packed in 20 centimeter length glass column. 25 mL extracted fraction poured on head of the packed glass column. Different proportions of Hexane/Ethyl acetate mixtures were used 90/10; 85/15; 80/20; 70/30; 75/25; 60/40; 50/50; 40/60; 30/70 for purification process. Hexane/Ethyl acetate fraction with 40/60 isocratic composition fraction showed high antimicrobial activity. 25ml fraction was further evaporated at 40°C temperature in rotate evaporator and reduced volume to 2.5ml. Initially peak checked on c18 column and gradient method optimized for separation of peak .C18 column with 0.1% formic acid in 1000 ml of water and acetonitrile/ methanol (1: 1) mixture mobile phase used for HPLC gradient studies [10].

## Results and Discussion:

Actinobacteria, the prolific producers of bioactive metabolites and antibiotics are important suppliers for pharmaceutical industry. NLKB 45 strain belonging to the Streptomyces genus was isolated from Nellore coastal region. The strain was subjected to fermentation in starch casein broth medium. The ethyl acetate extracts of this strain show excellent antimicrobial activity against *Salmonella sp*, *Staphylococcus aureus*, *E. coli*, and *B. subtilus*. IC50 value of extracted the compound from NLKPB45 showed 18.38 ug/ml against DA MB 231 cell line [6,7] In the present study extracted the compound subjected to NMR studies for structural elucidation.

## Semi-preparative HPLC optimized method conditions:

**Figure 1:**
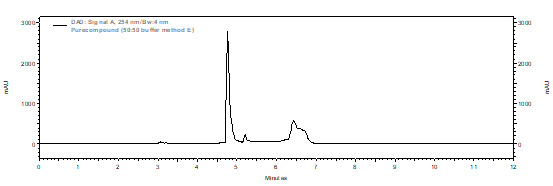
Chromatogram of the compound

The Agilent 1260 infinity series HPLC system was used. The Zorbax SB-C18 (100 mm x 9.4 mm, 5 µm, Agilent Technologies, USA) column was used for the method development and method isolation studies. 0.1% formic acid in 1000 mL of water used as mobile phase A and acetonitrile/ methanol (1: 1) mixture used as mobile phase B. solvents were filtered through 0.45 µm membrane filter (Millipore PVDF) and degassed in ultrasonic bath for 10 mins. The flow rate and injection volumes were used 1.0 mL min-1 and 10 µL, respectively. Dissolved in mobile phase (A:B-70:30) of HPLC (Figure 1). Initially scouting run given on reverse phase C18 column and gradient method was optimized for separation of peak. The analysis was carried out under gradient conditions as follows: time (minute)/% mobile phase-B; 0/30, 7/100, 10/100, 11/30, 12/30. Chromatographic data were acquired at 254 nm and processed using EZ chrome OpenLab software version A.04.05 as data handling system. Chromatogram showing well seperation of the compound and no interference with the other sample components.

## FTIR analysis:

Fine grained mixture of about 2 mg of sample and 200 mg of dried potassium bromide (KBr) in a mortar and pestle. Mix it thoroughly in mortar and pestle. Spread it, uniformly in a suitable die compress under vacuum to form a pellet. Transfer a pellet in a sample holder and take IR spectrum recorded from 4000 cm-1 to 400 cm-1. This the compound subjected to FTIR analysis for the identification of major functional groups (Table 1). FTIR spectra showed presence of N-H, O-H, C=C, C-O stretching vibrations observed showed in (Figure 1).

## Mass spectrometry study:

**Figure 3:**
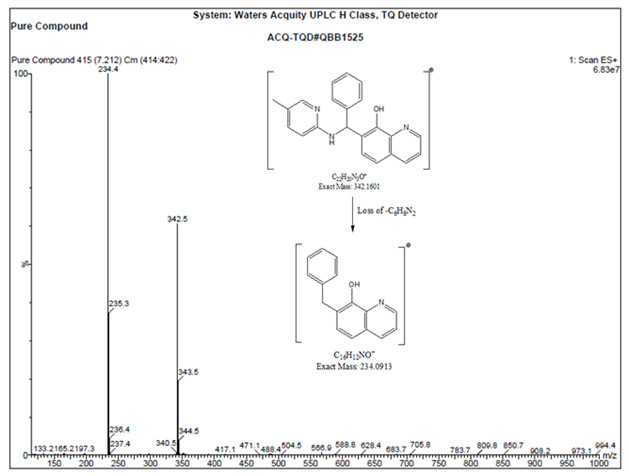
Mass spectrum of the compound (+ve ion mode)

The Waters Acquity UPLC-MS with TQD detector system was used. The C18 (100 mm x 2.1 mm, 1.7 µm, Agilent Technologies, USA) column was used for the mass studies. 0.1% formic acid in 1000 mL of water used as mobile phase A and acetonitrile used as mobile phase B. solvents were filtered through 0.45 µm membrane filter (Millipore PVDF) and degassed in ultrasonic bath for 10 mins. The flow rate and injection volumes were used 0.35 mL/min−1 and 5 µL, respectively. The analysis was carried out under gradient conditions as follows: time (minute)/% mobile phase-B; 0/5, 2/5, 8/95, 12/95, 12/5, 15/5. Mass data were acquired at 254 nm and processed using Mass Lynx software as data handling system. MS tune parameters capillary voltage 3.5, Cone voltage 3.0V, Source temperature 120°C and Desolvation temperature 350°C. Mass spectrum of the compound (+ve ion mode) observed as 342.5 as shown in Figure 3.

## NMR studies:

**Figure 4:**
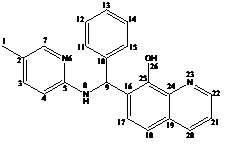
Structure of the compound

1D/2D NMR spectra (1H-NMR, 13C-NMR, DEPT-135, COSY and HSQC) of the compound were recorded on Bruker 400 MHz spectrometer equipped with superconducting magnet and 5 mm NMR probe .System was controlled by Topspin (version 3.5). For 1H- NMR 5mg compound suspended in 600µL of Di-methyl sulfoxide (DMSO-d6) obtained from Sigma-Aldrich Co. (St. Louis, MO, USA). NMR experiments like 13C, DEPT and 2D NMR carried out with 15mg of the compound to depict the structure (Figure 4).

## 1H-NMR technique:

**Figure 5:**
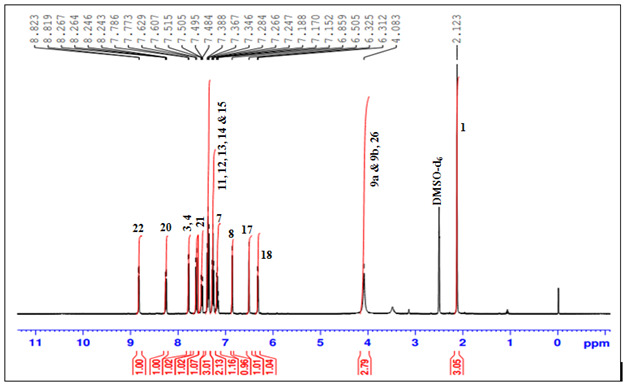
1H-NMR Spectrum of unknown compound

Peak assignments and multiplicities for the the compound were made based on the 1H-NMR, 13C-NMR and DEPT 135 connectivity. The prominent signals of selected solvent, i.e., DMSO-d6 arose at δ 2.54 ppm and δ 39.98 ppm in 1H NMR and 13C NMR, respectively. 1H-NMR spectrum showed total 19 protons in the unknown compound structure. Figure 5 shows 1H NMR peak assignments of the compound. The notable signals included: i) One singlet arose at δ 2.123 ppm corresponding to methyl protons (H-1); ii) One methine proton observed at δ 7.607 to 7.629 ppm (H-3); iii) One methine proton observed at δ 7.773 to 7.786 ppm (H-4); iv) One methine proton observed at δ 7.152 to 7.188 ppm (H-7); v) One amine (H-8) and methine proton observed at δ 6.859 ppm vi) One methine proton observed at δ 4.083 ppm (H-9); vii) Five methine protons arose at δ 7.247 ppm to δ 7.388 ppm (H-11, H-12, H-13, H-14 and H-15) corresponding to phenyl ring; viii) One methine proton observed at δ 6.505 ppm (H-17), corresponding to quinoline ring moiety; ix) One methine proton observed at δ 6.312 to 6.325 ppm (H-18), corresponding to quinoline ring moiety; x) One methine proton observed at δ 8.243 to 8.267 ppm (H-20), corresponding to quinoline ring moiety; xi) One methine proton observed at δ 7.484 to 7.515 ppm (H-21), corresponding to quinoline ring moiety; xii) One methine proton observed at δ 8.819 to 8.829 ppm (H-22), corresponding to quinoline ring moiety; xiii) One proton arose at δ 2.123 ppm (H-26) clearly shown in Table 2.

## 13C-NMR technique:

**Figure 6:**
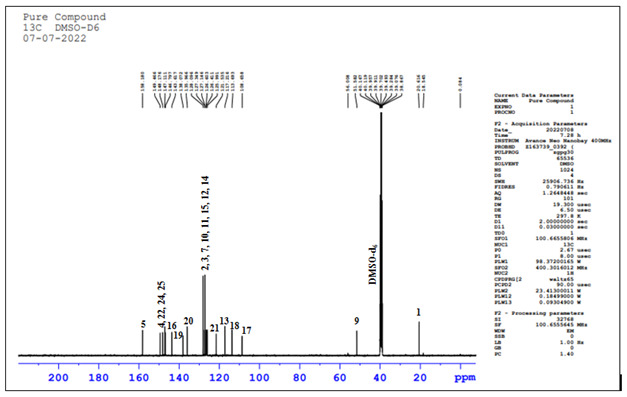
13C-NMR Spectrum of the compound

In Figure 6, 13C-NMR spectrum showed total 22 carbon atoms in the the compound structure. The 13C NMR signals include; i) One methyl carbon observed at δ 20.78 ppm corresponding to methyl carbon (C-1); ii) Two quaternary carbons and six methine carbons arose at δ 126.227 ppm to 128.535 ppm corresponding to C-2, C3, C-7, C-10, C-11, C-12, C-13, C-14 and C-15 positions; iii) Two methine carbon atoms observed at δ 146.797 ppm to 147.111 ppm corresponding to C-4 and C-24 positions; iv) One quaternary carbon arose at δ 158.180 ppm corresponding to C-5 position; vi) One methine carbon arose at δ 56.008 ppm corresponding to C-9 position; vii) One methine carbon observed at δ 117.216 ppm corresponding to C-13 position; viii) One quaternary carbon arose at δ 143.617 ppm corresponding to C-16 position; ix) One methine carbon observed at δ 108.658 ppm (C-17); x) One methine carbon arose at δ 113.693 ppm corresponding to C-18 position; xi) One quaternary carbon arose at δ 138.072 ppm corresponding to C-19 position; x) One methine carbon arose at δ 135.966 ppm corresponding to C-20 position; x) One methine carbon arose at δ 121.535 ppm corresponding to C-21 position; x) One methine carbon arose at δ 148.176 ppm corresponding to C-22 position; xi) One quaternary carbon arose at δ 146.797 ppm to 147.111 corresponding to C-24 position; xi) One quaternary carbon arose at δ 149.466 ppm corresponding to C-25 position shown in Table 3.

## 13C-NMR and DEPT-135 NMR:

**Figure 7:**
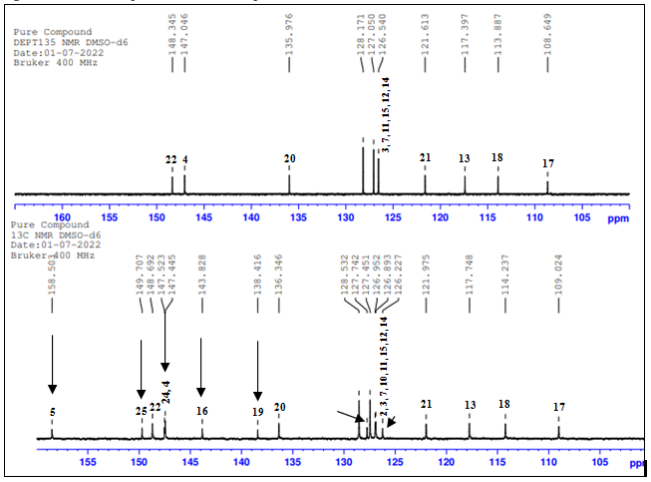
13C and DEPT-135 NMR Spectrum overlay

In Figure 7, 13C-NMR and DEPT-135 data has given information about the methyl, methine and quaternary carbon atoms in the the compound structure. DEPT-135 spectrum quaternary carbon signals were absent. We have observed these signals in 13C NMR spectrum. So, there is no inverted peak in DEPT-135 spectrum which indicates absence of methylene carbon atoms in the the compound structure. Methyl protons were observed δ 20.89 ppm in DEPT-135 spectrum (Table 4).

## 1H-1H COSY technique (2D):

**Figure 8:**
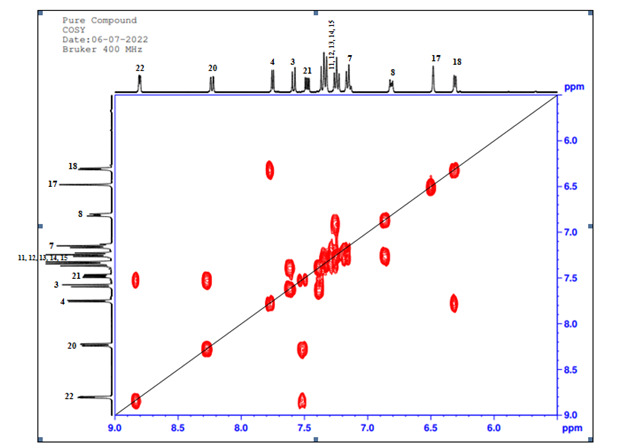
COSY NMR (2D) Spectrum of unknown compound

Table 5 shows proton-proton correlations within the the compound structure. COSY is a 1H-1H Homonuclear shift correlation spectrum (Figure 8) which contains information on spin coupling networks within a constituent residue through the observation of cross peaks off the diagonal. The strategy of assigning a COSY spectrum is to find one unmistakably characteristic signal from which to begin the tracing of a spin system or network. 1H signal correlation spectroscopy correlates proton that are three bonds away.

## 1H-13C HSQC technique:

**Figure 9:**
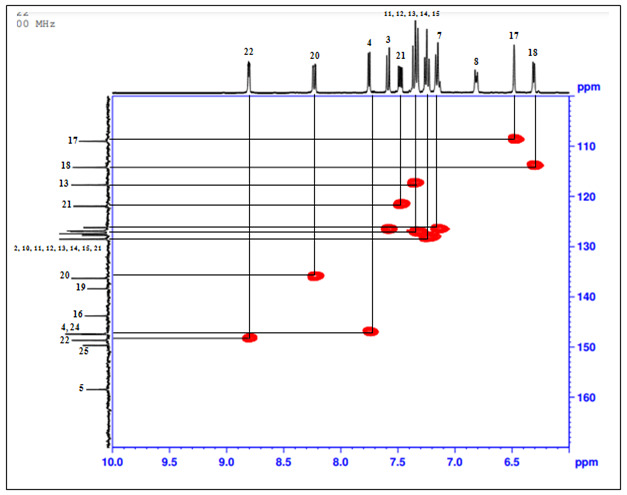
HSQC NMR Spectrum of the compound

Table 6 shows Heteronuclear single quantum correlation spectroscopy (HSQC) is a 2D experiment. It correlates the chemical shift of proton with the chemical shift of the directly bonded carbon. It utilizes the one bond coupling between carbon (13C) and proton (1H) as shown in Figure 9.

## Conclusion:

The antimicrobial compound extracted from NLKB 45 was purified and isolated. Analysis was done using FTIR, Mass spectrometry and NMR spectroscopy. The isolated compound was identified as 7-((5-methylpyridin-2-ylamino) (phenyl) methyl) quinolin-8-ol with the following structure.

## Figures and Tables

**Figure 2:**
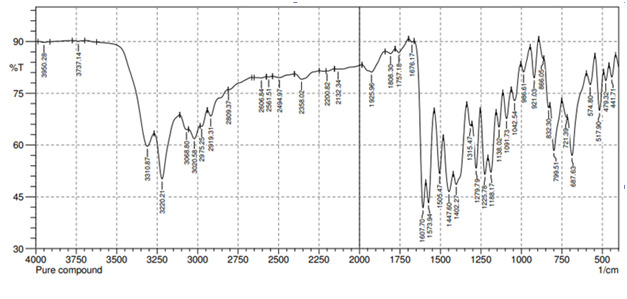
FTIR Spectrum of the compound

**Table 1:** Observed functional groups of unknown compound

**Functional group**	**Frequency (cm^-1^)**
N-H Stretching	3310.87
O-H Stretching	3220.21
C=C Aromatic stretching	1607.7
C-O Stretching	1225.78

**Table 2:** Analysis of the 1H-NMR spectral assignments

**Position**	**Protons**	**Chemical shift (ppm)**	**Multiplicity**	**Integral**
1	CH3	2.123	s	3.05
3	CH	7.607 to 7.629	d	1.02
4	CH	7.773 to 7.786	d	1.02
7	CH	7.152 to 7.188	t	1.16
8	NH	6.859	s	0.96
9	CH (a & b)	4.083	s	2.79
11	CH	7.247 to 7.388	t	3.01
12	CH	7.247 to 7.388	t	2.13
13	CH	7.247 to 7.388	t	3.01
14	CH	7.247 to 7.388	t	2.13
15	CH	7.247 to 7.388	t	3.01
17	CH	6.505	s	1.01
18	CH	6.312 to 6.325	d	1.04
20	CH	8.243 to 8.267	d	1
21	CH	7.484 to 7.515	d	1.07
22	CH	8.819 to 8.829	d	1
26	OH	2.123	s	3.05

**Table 3:** Analysis of the 13C-NMR spectral assignments

**Position**	**Carbons**	**Chemical shift (ppm)**
1	CH3	20.78
2	C	126.227 to 128.535
3	CH	126.227 to 128.535
4	CH	146.797 to 147.111
5	C	158.18
6	CH	126.227 to 128.535
9	CH	56.008
10	C	126.227 to 128.535
11	CH	126.227 to 128.535
12	CH	126.227 to 128.535
13	CH	117.216
14	CH	126.227 to 128.535
15	CH	126.227 to 128.535
16	C	143.617
17	CH	108.658
18	CH	113.693
19	C	138.072
20	CH	135.966
21	CH	121.535
22	CH	148.176
24	C	146.797 to 147.111
25	C	149.466

**Table 4:** Analysis of the 13C and 135-NMR Spectrum

**Position**	**Carbons**	**^13^C (δppm)**	**DEPT-135 (δppm)**
1	CH3	20.78	20.89
2	C	126.227 to 128.535	-
3	CH	126.227 to 128.535	126.540 to 128.171
4	CH	146.797 to 147.111	147.046
5	C	158.18	-
6	CH	126.227 to 128.535	126.540 to 128.171
7	CH	56.008	52.15
8	C	126.227 to 128.535	-
9	CH	126.227 to 128.535	126.540 to 128.171
10	CH	126.227 to 128.535	126.540 to 128.171
11	CH	117.216	117.397
12	CH	126.227 to 128.535	126.540 to 128.171
13	CH	126.227 to 128.535	126.540 to 128.171
14	C	143.617	-
15	CH	108.658	108.649
16	CH	113.693	113.887
17	C	138.072	-
18	CH	135.966	135.346
19	CH	121.535	121.975
20	CH	148.176	148.345
21	C	146.797 to 147.111	-
22	C	149.466	-

**Table 5:** Analysis of COSY-NMR correlations

**Position**	**Protons**
3	H-4
4	H-3
8	H-9
9	H-8
11	H-12
12	H-11, H-13
13	H-12, H-14
14	H-13, H-15
15	H-14
17	H-18
18	H-17
20	H-21
21	H-20, H-22
22	H-21

**Table 6:** Analysis of HSQC-NMR peak assignments

**Position**	**Protons**
1	C-1
3	C-3
4	C-4
7	C-7
9	C-9
11	C-11
12	C-12
13	C-13
14	C-14
15	C-15
17	C-17
18	C-18
20	C-20
21	C-21
22	C-22
